# Reevaluating the association between cholecystectomy and gastroesophageal reflux disease: recommendations for enhanced Mendelian randomization analysis

**DOI:** 10.1097/MS9.0000000000002541

**Published:** 2024-09-10

**Authors:** Xingcheng Zhu, Shi Fu, Junhao Chen

**Affiliations:** a Department of Clinical Laboratory, The Second People’s Hospital of Qujing City Qujing; b Department of Urology, The Second Affiliated Hospital of Kunming Medical University, Kunming, Yunnan Province, People’s Republic of China

Highlights

- Hand surgery as an exposure in Mendelian randomization (MR) is problematic due to significant heterogeneity. Conclusions based on a single disease outcome database may lack reliability. To enhance the robustness of the conclusions, integrating gastroesophageal reflux disease (GERD) results from multiple databases and performing a meta-analysis is advisable.
- Sensitivity analyses should incorporate MR-PRESSO methods, and when heterogeneity is present, the RadialMR package should be used to further exclude outlier SNPs.
- After conducting a two-sample Mendelian randomization analysis, it is essential to apply false discovery rate (Bonferroni) correction and Bayesian weighted Mendelian randomization to reduce the risk of false positives. Reverse MR validation is also needed to confirm the absence of bidirectional causality.
- Further validation can be achieved by incorporating NHANES database mining and transcriptomic analysis for triangulation.

*Dear Editor,*

We have carefully reviewed the article by Qian *et al*.^1^ published in the *International Journal of Surgery*, which utilized MR to assess the causal relationship between cholecystectomy and GERD. This finding contributes to our understanding of GERD’s pathogenesis and prevention. While we commend the authors for their excellent work and innovative ideas, we have several points we would like to discuss.

Despite the effective use of MR to estimate causal effects between exposure and outcome using genetic variants as instrumental variables, relying solely on a single disease outcome database may introduce limitations and biases. This could interfere with accurate causal inference. Hand surgery as an exposure in MR is particularly problematic due to significant heterogeneity, such as variations in hospital conditions, surgical methods, and surgeon expertise. These factors, including the use of robotic surgery, open surgery, and laparoscopic surgery, introduce considerable heterogeneity, undermining the reliability of the conclusions. Consequently, MR studies typically avoid using surgery as an exposure variable. If surgery must be used as an exposure, it is advisable to incorporate GERD results from other databases, perform separate MR analyses and then conduct a meta-analysis. For example, the FINN R10 database (gs://finngen-public-data-r10/summary_stats/finngen_R10_K11_REFLUX.gz) can be utilized. Integrating results from multiple databases can enhance the robustness and reliability of the conclusions^2^.

Secondly, the authors observed heterogeneity in their results, which was consistent with the significant heterogeneity associated with surgery. To effectively address this, the authors should incorporate MR-PRESSO tests and use the RadialMR package to detect and exclude outlier SNPs before re-conducting MR analyses. The MR-PRESSO method helps identify and correct potential pleiotropic bias, while the RadialMR package aids in excluding SNPs with high heterogeneity. This approach can significantly remove most outlier SNPs and improve the reliability of the results. Additionally, these methods provide further verification of the study’s robustness by detecting and addressing pleiotropy.

After performing a two-sample Mendelian randomization analysis, it is necessary to implement the False Discovery Rate (Bonferroni) correction and the Bayesian Weighted method to reduce the risk of false positives. The Bonferroni correction strictly controls for false positive results due to multiple tests, ensuring the credibility of significant results. Bayesian Weighted MR calculates the posterior distribution of each instrumental variable and weights the different instrumental variables based on these distributions. This weighting mechanism can reduce the impact of confounding instrumental variables and obtain a more robust causal effect estimate through weighted averaging. Additionally, reverse MR verification can confirm the unidirectionality of the causal relationship, ensuring the absence of bidirectional causality thereby enhancing the robustness and credibility of the conclusions^3,4^.

Additionally, NHANES database mining and transcriptomic analysis can be incorporated for further triangulation. The NHANES database includes GERD data, and the authors found that rs11887534 is significant for the outcome. Beyond this SNP, SNPs identified from two-sample MR can be converted to genes using the tool (https://biit.cs.ut.ee/gprofiler) and further analyzed with the GEO database for transcriptomic analysis. This approach allows for triangulation using MR, NHANES data mining, and GEO, enhancing the reliability and robustness of the study results from multiple perspectives^5^.

In conclusion, we express our gratitude and admiration for the contributions made by Qian *et al*. Our suggestions and concerns aim to further optimize the research methods, promoting a deeper understanding and development of therapeutic strategies in future studies.

## Ethical approval

Not applicable.

## Consent

Not applicable.

## Source of funding

Not applicable.

## Author contribution

X.Z.: design, literature search, and writing; S.F.: literature search and data collections; J.C.: providing language polish.

## Conflicts of interest disclosure

The authors declare no conflicts of interest.

## Research registration unique identifying number (UIN)

Not applicable.

## Guarantor

Junhao Chen.

## Data availability statement

All data generated and analyzed during this study are included in this published article. The data presented in the article may be requested by consulting the correspondence author.

## Provenance and peer review

Not applicable.
